# Should we marry a pharmacist? With or without separate property

**DOI:** 10.1186/1757-7241-23-S1-A39

**Published:** 2015-07-16

**Authors:** Elisa Worthington, Mette Juul-Gregersen

**Affiliations:** 1 Akut og Traumecenter, Ålborg Universitetshospital (AAUH), Aalborg, Denmark

## Background

In health care, there are increasing demands for efficiency and safe patient care. Concomitantly, requirements to patient-related documentation have risen among others for medication. Documentation is, however, time consuming and therefore not always done diligently, perhaps especially on weekends. One proven way to improve patient safety is the review of medication by a pharmacist after admission to the hospital to ensure correct medication (and documentation). Should we accept that pharmacists perform a medication review for us?

## Material and methods

From May 1 to May 31, 2014, we did a non-randomized retrospective study of 259 consecutive surgical patients admitted to the Emergency & Trauma Center, AAUH. The focus was on medication review (MG), aligned the medication (MA), medication status (MS) and registration of medical allergies (CAVE registration) at admission and at discharge.

## Results

At admission MG was made in 41.7% of the cases, MA of 40.5%, MS 75.7%, and in 17% of patients no medication review was performed. On discharge MG was made in 24.3% of the cases, MA 36.3%, MS 34.7%, and in 43.6% no review. In a total of 24 patients (9.3%), no review whatsoever of medicine was performed, neither in the ED nor throughout the in-hospital stay. Twelve cases (50%) occurred between Friday and Sunday. CAVE registration did not take place in 37 patients (14.3%). Of these 48.6% (18 cases), happened in the weekend.

## Discussion

Paired medicine review, first by a doctor followed by a pharmaceutical can contribute to safer patient care. The pharmacist have the opportunity to do a systematic and critical medication review, without the same time constraints doctors have in a busy ED and ensure that the correct medication is documented. But is it fair that due to increased documentation requirements doctors have to get help?

## Conclusion

Systematical pharmaceutical medication review will result in better documentation. But are we, as doctors, ready to hand over medication review to pharmacists? We do not renounce prescription rights but ensure better outcomes for our patients.

